# P20 Scleritis as initial presenting feature in ANCA-associated vasculitis

**DOI:** 10.1093/rap/rkab068.019

**Published:** 2021-10-19

**Authors:** Subrahmanyam Peddasomayajula, VenkateshwarRao Kesharaju, Anisur Rahman

**Affiliations:** 1 Rheumatology, Broomfield Hospital, Chelmsford, United Kingdom; 2 Ophthalmology, Broomfield Hospital, Chelmsford, United Kingdom; 3 Rheumatology, UCL, London, United Kingdom

## Abstract

**Case report - Introduction:**

ANCA-associated vasculitis (AAV) encompasses the clinical entities of GPA, MPA, renal-limited vasculitis and eGPA. Even though well recognised and described in the medical literature, ocular manifestations in AAV are relatively uncommon (<20%) and may precede, present concomitantly with, or follow systemic manifestations. Our patient developed scleritis as the first manifestation of AAV and presented to the ophthalmology department. Within a few days, he developed systemic symptoms and subsequently severe and potentially life-threatening pulmonary haemorrhage. With collaborative working, he received appropriate treatments and made a good recovery.

**Case report - Case description:**

Our patient is a 36-year-old Indian gentleman, who presented to Broomfield Hospital ophthalmology department in February 2021 with a 1-week history of pain and redness involving the left eye. Diagnosis of anterior scleritis was made and he received dexamethasone 0.1% eye drops and later switched to prednisolone 60mg/day. Investigations are shown below (Table 1). Diagnosis of AAV was made and he came under the care of the rheumatology. By this time, he noticed fleeting but severe arthralgia. He received three pulses of I.V. methyl prednisolone and received first dose of 1000mg of rituximab and one pulse of IV cyclophosphamide. His haemoglobin dropped with reduced oxygen saturation of 88% on air. Repeat CT chest showed extensive pulmonary haemorrhage and he was admitted to ITU. He did not need intubation and was transferred to University College London Hospital. Following five plasma exchanges and high-dose prednisolone, CRP fell from 113 to 6.6 and saturation improved to 98% on air. He completed four rituximab infusions. By June 2021 his chest X-ray returned to normal.

1. Table of Investigations

**Case report - Discussion:**

This patient’s story highlights multiple clinical aspects of AAV. Published literature states that ocular manifestations as “initial” presentation of AAV are very uncommon (about 6%). The ophthalmologist requested the correct investigations including ANCA which helped to establish the diagnosis. Within a few weeks, the patient went on to develop other systemic manifestations which necessitated stepping-up the immune therapy. Rituximab was chosen for remission induction as it is now established as an alternative to cyclophosphamide. We discussed the case at virtual MDT of ENRAD (Eastern Network for Rare Autoimmune Disease) and got swift approval to use rituximab. Unfortunately, his clinical course was complicated with development of pulmonary haemorrhage which is potentially life-threatening. The PEXIVAS trial (Walsh et al NEJM 382; 622-31 (2020)) compared groups randomised to plasma exchange or no plasma exchange in addition to corticosteroids and either rituximab or cyclophosphamide. Outcomes were not different between the groups. However, Kronbichler et al (Nephrol Dial Transplantation 36; 227-31 [2021]) have argued that there was a trend towards better outcomes in a subgroup with alveolar haemorrhage and that plasma exchange may still have a role in such patients. Following five cycles of plasma exchange our patient made an excellent recovery from pulmonary haemorrhage which is very rewarding.

**Case report - Key learning points:**

Scleritis is an uncommon presenting feature of AAV and should prompt the physician to look for systemic symptoms and check for ANCA serology.

To recognise pulmonary haemorrhage as a potential life-threatening manifestation in a patient with AAV who drops haemoglobin.

Despite lack of strong clinical trial evidence, plasma exchange can be a very useful therapeutic tool.

Early recognition and initiation of immune therapy is crucial to induce remission.

Collaborative working with clinicians from different medical specialities is the key for improved patient outcomes.

**Table:** 

IgG IF ANCA	Positive (cytoplasmic pattern)
MPO	<0.3 IU/mL (0 – 3.4)
PR3	155 IU/mL (0 – 1.9)
ANA, Anti-CCP	Negative
Anti – GBM	Negative
HIV	Negative
Hepatitis B &C	Negative
Treponema Pallidum Antibody	Not detected
T-spot TB	Negative
Serum Creatinine	68µmol/L.
Urine microscopy	4 RBC with no Casts.
Urine Protein/Creatinine Ratio	48.5 mg/mmol (0-15)
Hb	136 later dropped to 99 and later to 88
CRP	192.3 (at peak)
ESR	83 (at peak)
Direct Coombs test	Negative
Cytomegalovirus IgG	Detected
EBV (Nuclear and capsid antigen) IgG	Detected
CT Head and Orbits	no evidence of Intra-orbital, intra (or) Extra-conal abnormality
CT chest with contrast	Multiple, bilateral, ill-defined lung lesions. Some of them show central cavitation with furthermore extensive dense consolidation at the Right Lower lobe

